# Aprepitant and Fosaprepitant for Preventing Nausea and Vomiting in Patients Receiving Highly Emetogenic Chemotherapy—A Real-World Study

**DOI:** 10.3390/curroncol33070381

**Published:** 2026-06-24

**Authors:** Beyza Ünlü, Hacer Demir, Sena Ece Davarcı, Yaşar Culha, Duygu Özaşkın, Fariz Emrah Özkan, Sedat Yıldız, Canan Yıldız, Meltem Baykara

**Affiliations:** 1 Department of Medical Oncology, Afyonkarahisar Health Sciences University, Afyonkarahisar 03030, Turkey; hacer.demir@afsu.edu.tr (H.D.); ece.davarci@afsu.edu.tr (S.E.D.); dygalan91@gmail.com (D.Ö.); emrah.ozkan@afsu.edu.tr (F.E.Ö.); sedat.yildiz@afsu.edu.tr (S.Y.); canan.yildiz@afsu.edu.tr (C.Y.); meltem.baykara@afsu.edu.tr (M.B.); 2 Department of Medical Oncology, Sivas Numune Hospital, Sivas 58060, Turkey; drjasar@hotmail.com

**Keywords:** chemotherapy-induced nausea and vomiting, aprepitant, fosaprepitant, antiemetic therapy, Neurokinin-1 receptor antagonist, supportive care

## Abstract

Chemotherapy-induced nausea and vomiting substantially impair patients’ quality of life despite considerable advances in supportive care. Neurokinin-1 receptor antagonists, including oral aprepitant and intravenous fosaprepitant, constitute essential components of antiemetic regimens for highly emetogenic chemotherapy. In this prospective, non-randomized observational study, we compared the efficacy of oral aprepitant and intravenous fosaprepitant administered in combination with 5-hydroxytryptamine-3 receptor antagonists and dexamethasone in 136 chemotherapy-naive patients receiving cisplatin- or doxorubicin–cyclophosphamide-based regimens. Complete response rates during the acute (0–24 h), delayed (24–120 h), and overall (0–120 h) phases were comparable between the two groups, with no statistically significant differences in emesis severity. Multivariable analyses further demonstrated similar effectiveness irrespective of age, sex, or chemotherapy regimen. These findings indicate that no statistically significant differences in antiemetic efficacy were observed between aprepitant and fosaprepitant in routine clinical practice. Fosaprepitant may therefore represent a practical alternative when oral administration is not feasible.

## Simple Summary

Chemotherapy-induced nausea and vomiting are important side effects that negatively affect the quality of life of patients receiving cancer treatment and also make treatment adherence more difficult. Aprepitant and fosaprepitant are antiemetic treatments recommended in current guidelines for patients receiving highly emetogenic chemotherapy. In this prospective real-world study, the effectiveness of oral aprepitant and intravenous fosaprepitant used in combination with standard antiemetic therapy was compared. Both treatments were found to provide similar effectiveness in controlling nausea and vomiting during the acute and delayed periods after chemotherapy. While our findings support the existing literature, they also show that fosaprepitant may be an alternative, especially in situations where oral medication use is difficult.

## 1. Introduction

Significant progress has been achieved in the management of chemotherapy-related toxicities in recent years. Nevertheless, chemotherapy-induced nausea and vomiting (CINV) remains one of the most common and distressing adverse effects associated with cancer treatment. CINV occurs in approximately 80% of patients receiving chemotherapy and can markedly compromise patients’ quality of life, potentially leading to reluctance or refusal to continue treatment [1]. Clinical manifestations range from mild nausea to severe retching and vomiting [2]. CINV is classified into acute, delayed, anticipatory, breakthrough, and refractory emesis based on the timing and response to treatment [3].

Nausea and vomiting can be effectively controlled with appropriate antiemetic therapy. Among currently available agents, 5-hydroxytryptamine-3 (5-HT3) receptor antagonists are considered first-line therapy for patients receiving moderately or highly emetogenic chemotherapy (HEC) regimens [4]. The combination of 5-HT3 receptor antagonists with dexamethasone and NK1 receptor antagonists has been shown to improve the control of both acute and delayed emesis [5].

Aprepitant and fosaprepitant, both NK1 receptor antagonists prevent emesis by inhibiting the binding of substance P to NK1 receptors within the CNS [6]. These agents are used in combination with dexamethasone and 5-HT3 receptor antagonists for the prevention and management of both acute and delayed emesis. The recommended oral aprepitant regimen consists of 125 mg administered on day 1 of chemotherapy, followed by 80 mg on days 2 and 3 [7]. Fosaprepitant is a water-soluble prodrug of aprepitant. It was approved by both the Food and Drug Administration and the European Medicines Agency in January 2008 for the prevention of CINV associated with highly and moderately emetogenic chemotherapy. It is rapidly converted to aprepitant within approximately 30 min after intravenous administration [8]. Their pharmacologic characteristics and clinical efficacy in the prevention of CINV have been comprehensively reviewed previously [9].

Although both oral aprepitant and intravenous fosaprepitant are recommended as equivalent NK1 receptor antagonists in current antiemetic guidelines, prospective real-world comparative data remain limited, particularly in direct comparisons of the two agents across different patient populations [10,11]. In the present study, we evaluated the antiemetic efficacy of intravenous fosaprepitant versus oral aprepitant, each administered in combination with dexamethasone and a 5-HT3 receptor antagonist, in patients receiving HEC regimens, including cisplatin (>50 mg/m^2^) or doxorubicin (60 mg/m^2^) plus cyclophosphamide (600 mg/m^2^). The primary objective of the study was to compare the efficacy of these two NK1 receptor antagonist-based regimens in preventing CINV.

## 2. Materials and Methods

This single-center, prospective, non-randomized, controlled observational study was conducted between April 2022 and April 2023 at the Department of Medical Oncology, Afyonkarahisar Health Sciences University. Patients aged ≥18 years with an Eastern Cooperative Oncology Group performance status of 0–1, who were chemotherapy-naive and had adequate hematologic, renal, and hepatic function, were eligible for inclusion. All enrolled patients received HEC regimens consisting of cisplatin at doses >50 mg/m^2^ or a combination regimen of doxorubicin (60 mg/m^2^) and cyclophosphamide (600 mg/m^2^). Patients were excluded if they had previously received chemotherapy, had used antiemetic medications within 48 h before chemotherapy administration, were pregnant, had a known hypersensitivity to granisetron or dexamethasone, or could not be contacted for follow-up evaluations.

All patients received standard antiemetic prophylaxis on day 1 of chemotherapy, consisting of intravenous granisetron 3 mg as the 5-HT3 receptor antagonist and intravenous dexamethasone 8 mg as the corticosteroid component. No scheduled dexamethasone was administered beyond day 1. In addition to this background regimen, patients received one of two NK1 receptor antagonist protocols. Patients in the aprepitant group received oral aprepitant at a dose of 125 mg on day 1 of chemotherapy, followed by 80 mg on days 2 and 3. Patients assigned to the fosaprepitant group received a single intravenous dose of fosaprepitant 150 mg on day 1 only. The selection of the NK1 receptor antagonist regimen was determined by the treating physician according to clinical considerations, including the patient’s ability to tolerate oral medications, venous access status, and institutional formulary availability at the time of treatment.

CINV were evaluated through telephone interviews conducted at 24 h (acute phase) and 120 h (delayed phase) after chemotherapy administration. The severity of nausea and vomiting was graded according to the Common Terminology Criteria for Adverse Events (CTCAE), version 5.0. Nausea and vomiting severity scores reported in the study correspond to CTCAE v5.0 grading of symptoms collected during these telephone interviews. Complete response (CR) was defined as the absence of nausea and vomiting without the need for rescue antiemetic therapy throughout the assessment period. Rescue antiemetic therapy was administered at the discretion of the treating physician when clinically indicated, most commonly with metoclopramide or granisetron. Only data obtained from the first chemotherapy cycle were included in the final analysis.

Descriptive statistical methods were used to summarize demographic and clinical characteristics. Continuous variables were expressed as mean ± standard deviation (SD) or median (range), as appropriate, whereas categorical variables were presented as frequencies and percentages. The normality of continuous variables was evaluated using the Shapiro–Wilk test. The primary endpoint was overall CR (0–120 h). Secondary endpoints included acute CR (0–24 h) and delayed CR (24–120 h). Categorical variables were compared between treatment groups using the chi-square test or Fisher’s exact test, as appropriate. Continuous variables were analyzed using Student’s t-test or the Mann–Whitney U test according to the results of the normality assessment. Multivariable logistic regression analyses were performed to estimate adjusted odds ratios (ORs) and 95% confidence intervals (CIs) for the association between antiemetic regimen and CR. The models were adjusted for potential confounding variables, including age (continuous variable), sex (male vs. female), and chemotherapy regimen (cisplatin-containing vs. doxorubicin–cyclophosphamide-based therapy). Subgroup analyses were conducted using stratified analyses, and treatment-by-subgroup interaction effects were evaluated with likelihood ratio tests.

Emesis severity scores were compared between the aprepitant and fosaprepitant groups using the Mann–Whitney U test because the data did not satisfy the assumptions of normal distribution. Mean rank values were calculated for each treatment group, and effect size (r) was estimated to determine the magnitude of differences between groups. All statistical tests were two-sided, and a *p*-value < 0.05 was considered statistically significant. Statistical analyses were performed using SPSS software version 20 (IBM Corp., Armonk, NY, USA).

## 3. Results

### 3.1. Study Population and Baseline Characteristics

Between April 2022 and April 2023, a total of 136 patients receiving HEC were enrolled in this prospective observational study. Of these, 77 patients (56.6%) received oral aprepitant, while 59 patients (43.4%) received intravenous fosaprepitant as part of their antiemetic prophylaxis regimen. The mean age of the study population was 58.1 ± 11.1 years (median: 60 years; range: 20–79 years). The majority of patients were aged ≥55 years (n = 88, 64.7%). Female patients accounted for 56.6% of the cohort (n = 77). With regard to chemotherapy regimens, 59 patients (43.4%) received doxorubicin–cyclophosphamide (AC)-based regimens, whereas 77 patients (56.6%) received cisplatin-based chemotherapy. The most common underlying malignancy was breast cancer, observed in 74 patients (54.4%). This was followed by lung cancer (n = 20, 14.7%), genitourinary malignancies (n = 14, 10.3%), and gastrointestinal, hepatobiliary, and pancreatic cancers (n = 10, 7.4%) (Table 1).

Baseline characteristics were comparable between the two treatment groups, with no statistically significant differences observed in mean age (aprepitant: 58.6 ± 11.6 years vs. fosaprepitant: 57.3 ± 10.6 years; *p* = 0.507), age distribution (*p* = 0.544), sex distribution (*p* = 0.888), or chemotherapy regimen distribution (*p* = 0.888).

### 3.2. Response Outcomes

**Figure 1:**
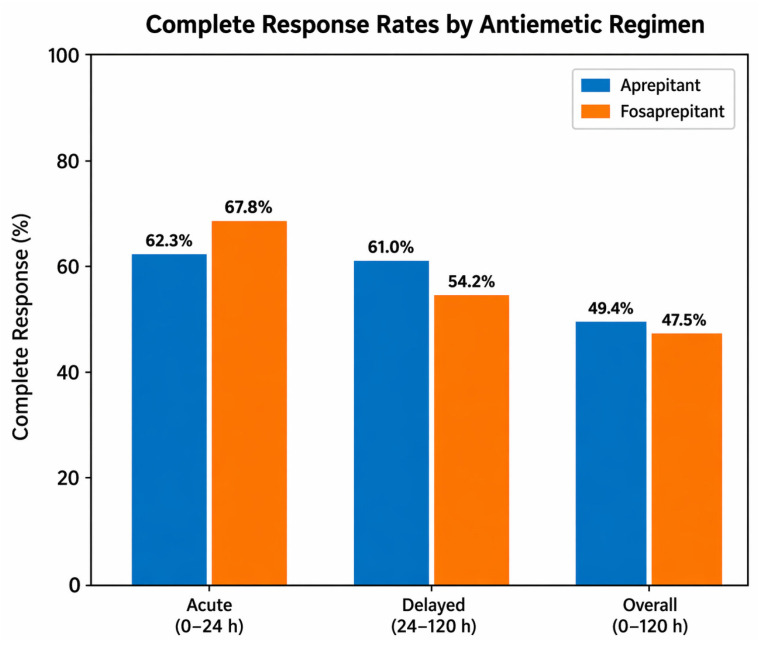
Comparison of complete response (CR) rates between the aprepitant and fosaprepitant groups during the acute (0–24 h), delayed (24–120 h), and overall (0–120 h) phases following highly emetogenic chemotherapy (HEC). CR was defined as the absence of nausea and vomiting and no requirement for rescue antiemetic therapy. Complete response rates were 62.3% (95% CI, 51.5–73.2) and 67.8% (95% CI, 55.9–79.7) during the acute phase, 61.0% (95% CI, 50.1–71.9) and 54.2% (95% CI, 41.5–66.9) during the delayed phase, and 49.4% (95% CI, 38.2–60.5) and 47.5% (95% CI, 34.7–60.2) during the overall phase for the aprepitant and fosaprepitant groups, respectively. CR rates were comparable between the two treatment groups across all phases, with no statistically significant differences observed.

In the acute phase (0–24 h), CR rates were similar between the aprepitant and fosaprepitant groups (62.3% vs. 67.8%, *p* = 0.509). Likewise, delayed phase (24–120 h) CR rates did not differ significantly between the two treatment arms (61.0% vs. 54.2%, *p* = 0.426). Overall CR rates (0–120 h) were 49.4% in the aprepitant group and 47.5% in the fosaprepitant group (*p* = 0.827). These results indicate comparable antiemetic efficacy between fosaprepitant and aprepitant in patients receiving HEC (Figure 1).

### 3.3. Factors Associated with CR

In univariate analyses, neither acute nor overall CR rates differed significantly according to sex or age category. Chemotherapy regimen was not significantly associated with acute CR (*p* = 0.250). In univariate analyses, delayed complete response rates appeared lower among patients receiving AC-based chemotherapy compared with cisplatin-based regimens. However, this association was attenuated and no longer statistically significant after adjustment for potential confounders in the multivariable model. Therefore, this finding should be considered exploratory and hypothesis-generating rather than conclusive. For overall CR, a borderline reduction in response was observed in the AC group compared with the cisplatin group (39.0% vs. 55.8%; *p* = 0.051; OR: 1.98; 95% CI: 0.99–3.95) (Table 2).

### 3.4. Multivariable Analysis

Multivariable logistic regression analyses adjusted for age, sex, and chemotherapy regimen showed that the antiemetic regimen (fosaprepitant vs. aprepitant) was not independently associated with acute, delayed, or overall CR. Similarly, age and sex were not independent predictors of treatment response in any phase. The association observed in the univariate analysis between chemotherapy regimen and delayed CR was not statistically significant in the multivariate analysis, nor was an independent association observed for acute or overall complete response. Overall, the antiemetic regimen was not independently associated with acute, delayed, or overall complete response in the adjusted analyses, and no statistically significant differences were observed between the fosaprepitant and aprepitant groups. Adjusted odds ratios were estimated using aprepitant treatment, female sex, age <55 years, and AC chemotherapy as the reference categories (Table 2).

### 3.5. Subgroup Analyses

**Figure 2:**
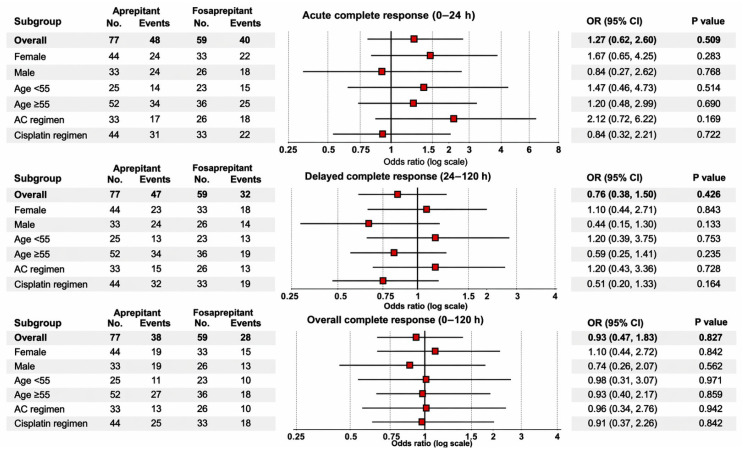
Subgroup analyses of CR rates in patients receiving aprepitant or fosaprepitant for the prevention of chemotherapy-induced nausea and vomiting (CINV). Forest plots present odds ratios (ORs) with 95% confidence intervals (CIs) for acute (0–24 h), delayed (24–120 h), and overall (0–120 h) CR across predefined subgroups, including sex, age (<55 vs. ≥55 years), and chemotherapy regimen (doxorubicin–cyclophosphamide [AC] vs. cisplatin-based regimens). Odds ratios are expressed relative to the aprepitant group (reference category); values greater than 1 indicate higher odds of CR with fosaprepitant, whereas values less than 1 indicate higher odds of CR with aprepitant. No statistically significant differences were observed between the two antiemetic regimens across all subgroups, and no evidence of treatment effect modification was identified.

Subgroup analyses were conducted according to sex, age group, and chemotherapy regimen for acute, delayed, and overall CR outcomes. Across all evaluated subgroups, fosaprepitant demonstrated efficacy comparable to that of aprepitant, with no statistically significant differences observed between treatment groups. The treatment effect remained consistent across sex, age categories, and chemotherapy regimens, and no evidence of effect modification was identified. Detailed results of the subgroup analyses are presented in Figure 2.

### 3.6. Emesis Severity Analysis

In the acute phase, emesis severity scores did not differ significantly between the aprepitant and fosaprepitant groups (Mann–Whitney U = 2180.5; *p* = 0.635). The mean rank values were 69.68 in the aprepitant group and 66.96 in the fosaprepitant group, and the effect size was negligible (r = 0.04). Similarly, in the delayed phase, no statistically significant difference was observed between the two groups with respect to emesis severity (Mann–Whitney U = 2035.0; *p* = 0.234). The mean rank values were 65.43 in the aprepitant group and 72.51 in the fosaprepitant group, and the effect size was small (r = 0.10). Overall, these findings indicate that emesis severity did not differ significantly between aprepitant and fosaprepitant during either the acute or delayed phases (Table 3).

## 4. Discussion

In this prospective observational study, we compared the efficacy of NK1 receptor antagonist-based regimens using real-world data in patients receiving HEC. No statistically significant differences were observed between aprepitant and fosaprepitant in terms of CR rates or emesis severity across the acute, delayed, and overall phases. These findings suggest that NK1 receptor antagonists provide comparable antiemetic efficacy in routine clinical practice.

Current clinical guidelines recommend NK1 receptor antagonist-based triple prophylaxis (NK1RA + 5-HT3 receptor antagonist + dexamethasone) for patients undergoing HEC [2,3,12]. Both oral aprepitant and intravenous fosaprepitant are recognized as acceptable NK1 receptor antagonist options, with no explicit preference between formulations. The real-world evidence from our study supports these recommendations by demonstrating that both agents exhibit similar clinical efficacy when administered in combination with standard antiemetic therapy.

The antiemetic efficacy of fosaprepitant was initially demonstrated in clinical studies conducted in the early 2000s. In contrast, the efficacy of aprepitant has been extensively established in randomized clinical trials, including the pivotal multinational study by Hesketh et al., which showed a significant improvement in CR rates among patients receiving HEC [13]. Cocquyt et al. reported CR rates of 37% in the acute phase and 48% in the delayed phase in patients receiving high-dose cisplatin who were treated with fosaprepitant in addition to standard antiemetic therapy [14]. Similarly, Van Belle et al. demonstrated acute and delayed CR rates of 36% and 46%, respectively, in patients receiving cisplatin-based chemotherapy treated with fosaprepitant plus dexamethasone [15]. Subsequently, randomized trials comparing fosaprepitant- and aprepitant-based regimens demonstrated bioequivalence and non-inferiority between intravenous fosaprepitant and standard oral aprepitant regimens. Large-scale studies involving patients receiving cisplatin-based chemotherapy confirmed that a single 150 mg intravenous dose of fosaprepitant combined with a 5-HT3 receptor antagonist and dexamethasone was non-inferior to a three-day oral aprepitant regimen in preventing both overall and delayed CINV [16,17,18,19]. In our study, CR rates in the acute phase were numerically higher in the fosaprepitant group, whereas higher response rates were observed in the aprepitant group during the delayed phase; however, these differences were not statistically significant.

Previous studies have identified female sex and younger age as important risk factors for CINV [20]. In the present study, although acute and delayed CR rates were numerically lower in female patients compared with male patients, this difference did not reach statistical significance. Similarly, no significant differences were observed across age groups. The relatively higher proportion of female patients and individuals aged >55 years in the study cohort may have influenced the statistical power and highlights the need for larger studies with more balanced subgroup distributions.

Subgroup analyses represent an important component of the present study. Analyses stratified by sex, age group, and chemotherapy regimen demonstrated no significant differences in efficacy between fosaprepitant and aprepitant in any subgroup. The consistency of the treatment effect across all subgroups suggests that both NK1 receptor antagonist formulations exhibit comparable clinical performance across diverse patient populations. Intravenous fosaprepitant may be particularly advantageous in patients who are unable to tolerate oral medications.

The CR rates observed in our study were relatively lower than those reported in some previous studies. This discrepancy may be attributed to differences in patient characteristics, real-world clinical conditions, and variations in adherence to antiemetic regimens. Although all patients received guideline-concordant triple antiemetic prophylaxis, factors such as chemotherapy regimen type, individual susceptibility to CINV, and differences in supportive care practices may have influenced the observed outcomes [21,22]. In addition, the higher proportion of female patients in the study population may have contributed to the lower response rates. Furthermore, differences in study design, including the observational nature of the present study and variations in outcome assessment methods, may also account for discrepancies with previously published literature.

A key strength of this study is the use of real-world data to compare the effectiveness of oral and intravenous NK1 receptor antagonists. Real-world studies typically include more heterogeneous patient populations than randomized clinical trials with strict eligibility criteria, thereby better reflecting routine clinical practice. In addition, separate evaluation of acute and delayed phases allowed for a time-dependent assessment of antiemetic efficacy. Adjustment for potential confounding factors through multivariable analyses further strengthened the robustness and reliability of the findings.

This study has several limitations that should be considered. The non-randomized observational design may have introduced potential selection bias. Because treatment allocation was determined by the treating physician according to clinical considerations, residual confounding by indication cannot be excluded. Patients unable to tolerate oral medications may differ systematically from those receiving oral therapy with respect to factors that could influence CINV outcomes. Due to the differing routes of administration (oral versus intravenous), blinding of patients and treating physicians was not feasible. However, to minimize assessment bias, outcome assessors who conducted telephone interviews at 24 and 120 h were blinded to the antiemetic regimen received by each patient. The single-center design may limit the generalizability of the findings to other clinical settings with different patient populations or antiemetic protocols. In addition, the absence of systematic collection of adverse event and safety data represents an important limitation, as does the lack of quality of life assessments alongside nausea and vomiting evaluation. The definition of complete response used in the present study also represents a potential limitation. Complete response was defined as the absence of both nausea and vomiting without the need for rescue antiemetic therapy. Because some previous studies have defined complete response primarily as no emesis and no rescue medication, differences in outcome definitions may have contributed to the relatively lower complete response rates observed in our cohort and may limit direct comparisons with the published literature [17]. Furthermore, several established patient-related risk factors for CINV, including history of motion sickness, pregnancy-related nausea and vomiting, alcohol consumption, anxiety, baseline nausea, and opioid use, were not systematically collected and therefore could not be included in the adjusted analyses. Furthermore, the observed complete response rate was lower than anticipated during the planning phase of the study, which may have reduced the statistical power to detect small differences between treatment groups. In addition, outcome assessment was based on patient-reported symptoms obtained through telephone interviews, which may have introduced recall bias or reporting inaccuracies. Although granisetron was used according to institutional practice during the study period, palonosetron is frequently preferred in contemporary antiemetic guidelines and clinical practice for patients receiving highly emetogenic chemotherapy. In addition, olanzapine was not routinely incorporated into antiemetic prophylaxis during the study period; therefore, the applicability of the findings to contemporary four-drug antiemetic regimens may be limited. Finally, although the sample size was sufficient for the primary analysis, statistical power for subgroup analyses, particularly within the AC cohort, was limited. Therefore, larger, multicenter studies are warranted to further evaluate potential effect modification according to age, sex, chemotherapy type, and other relevant risk factors.

## 5. Conclusions

In conclusion, this study demonstrated no statistically significant difference between oral aprepitant and intravenous fosaprepitant in the prevention of acute and delayed CINV in patients receiving HEC. Both NK1 receptor antagonist-based antiemetic regimens showed comparable outcomes in terms of CR rates and emesis severity across the acute, delayed, and overall phases. In multivariable analyses, no association was identified between antiemetic regimen, age, sex, or chemotherapy type and CR. These findings suggest that oral aprepitant and intravenous fosaprepitant demonstrated similar observed effectiveness in the prevention of acute and delayed CINV. However, because the present study was observational and was not designed to formally establish equivalence or non-inferiority, the results should be interpreted as showing no statistically significant differences between the two regimens rather than demonstrating interchangeability. Furthermore, adverse events and tolerability outcomes were not systematically collected; therefore, safety comparisons between the two regimens could not be performed. Larger multicenter prospective and randomized studies are warranted to confirm these findings and further evaluate comparative safety.

## Figures and Tables

**Table 1:** Demographic and baseline clinical characteristics of the study population.

Characteristic	Aprepitant (n = 77)	Fosaprepitant (n = 59)	Total (n = 136)	*p*-Value
**Age, Years**				0.507
Median	60	59	60	
<55 years, No. (%)	25 (32.5)	23 (39.0)	48 (35.3)	
≥55 years, No. (%)	52 (67.5)	36 (61.0)	88 (64.7)	
**Sex, No. (%)**				0.888
Male	33 (42.9)	26 (44.1)	59 (43.4)	
Female	44 (57.1)	33 (55.9)	77 (56.6)	
**Diagnosis, No. (%)**				0.238
Breast cancer	42 (54.5)	32 (54.2)	74 (54.4)	
Lung cancer	12 (15.6)	8 (13.6)	20 (14.7)	
Genitourinary cancers	8 (10.4)	6 (10.2)	14 (10.3)	
Gastrointestinal–hepatobiliary & pancreatic	6 (7.8)	4 (6.8)	10 (7.4)	
Head and neck cancer	2 (2.6)	2 (3.4)	4 (2.9)	
Other	7 (9.1)	7 (11.9)	14 (10.3)	
**Chemotherapy regimen, No. (%)**				0.888
Cisplatin-containing	44 (57.1)	33 (55.9)	77 (56.6)	
Doxorubicin–cyclophosphamide	33 (42.9)	26 (44.1)	59 (43.4)	

**Table 2:** Multivariable logistic regression analysis of factors associated with CR.

Outcome	Variable	Adjusted OR	95% CI	*p*-Value
Overall CR	Antiemetic regimen (fosaprepitant vs. aprepitant)	0.94	0.47–1.88	0.864
	Age	0.92	0.43–1.96	0.828
	Sex (male)	1.23	0.44–3.42	0.689
	Chemotherapy regimen (cisplatin vs. AC)	2.25	0.79–6.41	0.129
Acute CR	Antiemetic regimen (fosaprepitant vs. aprepitant)	1.29	0.63–2.65	0.493
	Age	0.83	0.38–1.80	0.639
	Sex	0.66	0.23–1.88	0.432
	Chemotherapy regimen (cisplatin vs. AC)	1.06	0.37–3.05	0.914
Delayed CR	Antiemetic regimen (fosaprepitant vs. aprepitant)	0.76	0.38–1.53	0.441
	Age	1.02	0.48–2.21	0.952
	Sex	1.29	0.43–3.81	0.650
	Chemotherapy regimen (cisplatin vs. AC)	2.64	0.88–7.91	0.083

The *p*-value corresponds to the Mann–Whitney U test utilized for comparing the ordinal severity scores based on mean ranks across the groups.

**Table 3:** Distribution and comparison of emesis severity in the acute and delayed phases.

Phase	Variable	Aprepitant (n = 77)	Fosaprepitant (n = 59)	*p*-Value
Acute (0–24 h)	Mean ± SD	0.47 ± 0.68	0.46 ± 0.77	0.635
	Grade 0 n (%)	48 (62.3%)	40 (67.8%)	
	Grade 1, n (%)	23 (29.9%)	13 (22.0%)	
	Grade 2, n (%)	5 (6.5%)	4 (6.8%)	
	Grade 3, n (%)	1 (1.3%)	2 (3.4%)	
Delayed (24–120 h)	Mean ± SD	0.42 ± 0.55	0.64 ± 0.87	0.234
	Grade 0 n (%)	47 (61.0%)	32 (54.2%)	
	Grade 1, n (%)	28 (36.4%)	20 (33.9%)	
	Grade 2, n (%)	2 (2.6%)	3 (5.1%)	
	Grade 3, n (%)	0 (0%)	4 (6.8%)	

## Data Availability

The datasets generated and/or analyzed during the current study are available from the corresponding author upon reasonable request.

## Abbreviations

CI	Confidence intervals
CINV	Chemotherapy-induced nausea and vomiting
CR	Complete response
CTCAE	Common Terminology Criteria for Adverse Events
HEC	Highly emetogenic chemotherapy
OR	Odds ratios
SD	Standard deviation
